# Epidemiology of Tuberculosis and Progress Toward Meeting Global Targets — Worldwide, 2019

**DOI:** 10.15585/mmwr.mm7012a4

**Published:** 2021-03-26

**Authors:** Rena Fukunaga, Philippe Glaziou, Jennifer B. Harris, Anand Date, Katherine Floyd, Tereza Kasaeva

**Affiliations:** ^1^Division of Global HIV and TB, Center for Global Health, CDC; ^2^Tuberculosis Monitoring and Evaluation, Global Tuberculosis Programme, World Health Organization, Geneva, Switzerland.

Although tuberculosis (TB) is curable and preventable, in 2019, TB remained the leading cause of death from a single infectious agent worldwide and the leading cause of death among persons living with HIV infection (*1*). The World Health Organization’s (WHO’s) End TB Strategy set ambitious targets for 2020, including a 20% reduction in TB incidence and a 35% reduction in the number of TB deaths compared with 2015, as well as zero TB-affected households facing catastrophic costs (defined as costs exceeding 20% of annual household income) (*2*). In addition, during the 2018 United Nations High-Level Meeting on TB (UNHLM-TB), all member states committed to setting 2018–2022 targets that included provision of TB treatment to 40 million persons and TB preventive treatment (TPT) to 30 million persons, including 6 million persons living with HIV infection and 24 million household contacts of patients with confirmed TB (4 million aged <5 years and 20 million aged ≥5 years) (*3*,*4*). Annual data reported to WHO by 215 countries and territories, supplemented by surveys assessing TB prevalence and patient costs in some countries, were used to estimate TB incidence, the number of persons accessing TB curative and preventive treatment, and the percentage of TB-affected households facing catastrophic costs (*1*). Globally, TB illness developed in an estimated 10 million persons in 2019, representing a decline in incidence of 2.3% from 2018 and 9% since 2015. An estimated 1.4 million TB-related deaths occurred, a decline of 7% from 2018 and 14% since 2015. Although progress has been made, the world is not on track to achieve the 2020 End TB Strategy incidence and mortality targets (*1*). Efforts to expand access to TB curative and preventive treatment need to be substantially amplified for UNHLM-TB 2022 targets to be met.

TB data are reported annually to WHO by 215 countries and territories and are reviewed and validated in collaboration with reporting entities (*1*). Four methods are used to estimate TB incidence: 1) TB prevalence surveys; 2) notifications from country surveillance systems, adjusted by a standard factor to account for underreporting, overdiagnosis, and underdiagnosis; 3) TB inventory studies that measure the level of underreporting of persons with diagnosed TB combined with capture-recapture modeling; and 4) national notification data supplemented by expert opinion regarding case detection gaps. For HIV-negative persons, estimates of TB mortality were based on all-cause mortality data from civil registration and vital statistics, mortality surveys, or the product of TB incidence and the case-fatality rate (CFR) (i.e., the proportion of persons with TB who die from the disease) (*1*). Among persons living with HIV infection, TB mortality estimates were calculated as the product of incidence and the CFR. The number of persons receiving TB curative and preventive treatment is reported by individual countries directly to WHO. National TB patient cost surveys were used to assess the proportion of TB-affected households facing catastrophic costs.

Globally, TB illness developed in an estimated 10 million persons in 2019 (130 per 100,000 population), 815,000 (8.2%) of whom were HIV-infected (Table). Overall, TB incidence declined 2.3% from 2018 and 9% from 2015. An estimated 1.4 million persons died from TB in 2019, including 208,000 persons who were living with HIV infection. The total number of TB deaths declined by 7% from 2018 to 2019 and by 14% since 2015 (*1*).

**TABLE:** Estimated number of incident tuberculosis (TB) cases, TB incidence rate, number of TB-associated deaths among all persons and among HIV-positive persons, and number of TB patients with rifampicin-resistant TB (RR-TB), by World Health Organization region — worldwide, 2019

WHO region	No. of TB cases, x1,000	Incidence*	No. of deaths, x1,000 (CFR, %)	No. of TB cases among HIV-positive persons, x1,000	No. of TB deaths among HIV-positive persons, x1,000	No. of RR-TB cases,^†^ x1,000	Incidence of RR-TB*^,†^	% of RR-TB cases^†^
**Global (all regions)**	**9,960**	**130**	**1,418 (14.2)**	**815.0**	**208.0**	**465**	**6.1**	**4.7**
African	2,470	226	547 (22.1)	595.0	169.0	77	7.0	3.1
Americas	290	29	22.9 (7.9)	29.0	5.9	11	1.0	3.8
Eastern Mediterranean	819	114	78.7 (9.6)	7.9	2.7	36	5.0	4.4
Europe	246	26	24.2 (9.8)	30.0	4.2	70	7.5	28.5
South-East Asia	4,340	217	652 (15.0)	117.0	20.0	171	8.6	3.9
Western Pacific	1,800	93	90.3 (5.0)	36.0	6.3	101	5.2	5.6

**Source**: Adapted from World Health Organization. Global tuberculosis report 2020. Geneva, Switzerland: World Health Organization; 2020. https://www.who.int/teams/global-tuberculosis-programme/tb-reports

**Abbreviation:** CFR = case-fatality rate.

* Number of cases per 100,000 population.

**^†^** Includes multidrug-resistant TB.

During 2019, multidrug-resistant (MDR) TB illness (TB that is resistant to at least isoniazid and rifampicin, the two most potent anti-TB drugs) (*5*) or rifampicin-resistant TB illness (RR-TB) developed in an estimated 465,000 persons. These patients accounted for 4.7% of all persons with TB, 3.3% of persons with a new TB diagnosis, and 18% of persons previously treated for TB.

Most persons who became ill with TB in 2019 lived in the WHO regions of South-East Asia (44%), Africa (25%), and the Western Pacific (18%), with smaller percentages in the Eastern Mediterranean (8.2%), the Americas (2.9%), and Europe (2.5%) (Table). Eight countries accounted for two thirds of the total global TB cases: India (26%), Indonesia (8.5%), China (8.4%), the Philippines (6.0%), Pakistan (5.7%), Nigeria (4.4%), Bangladesh (3.6%), and South Africa (3.6%). The WHO European and African regions have experienced the largest declines in incidence (19% and 16%, respectively) and mortality (31% and 19%, respectively) since 2015.

If persons who received a TB diagnosis that was reported to national authorities are assumed to be treated for TB (*1*), then in 2019, a total of 7.1 million persons were treated for TB, a slight increase from 7.0 million in 2018. With an estimated 10 million incident cases, this leaves a gap of 2.9 million persons with incident TB who either did not receive a diagnosis or did receive a diagnosis but were not reported to national authorities. Among the estimated 815,000 HIV-infected persons with cases of incident TB, 456,426 (56%) persons were reported as having received a diagnosis and been treated. Among the estimated 465,000 persons with incident MDR or RR-TB, only 177,099 (38%) were enrolled in MDR or RR-TB treatment.

A total of 4.1 million persons received TPT in 2019 (Figure), an 86% increase from 2.2 million in 2018 and a 300% increase from 1.0 million in 2015. Most persons who received TPT were persons living with HIV infection (3.5 million in 2019 and 1.8 million in 2018). Among the estimated 1.3 million children aged <5 years who were household contacts of TB patients, 433,156 (33%) received TPT in 2019, compared with 349,796 (27%) in 2018 (an 18% increase in the number of children treated). Among older household contacts, the number of persons who received TPT was 105,240 persons in 2019 and 73,811 in 2018 (a 43% increase). The total number of older household contacts is unknown.

**FIGURE:**
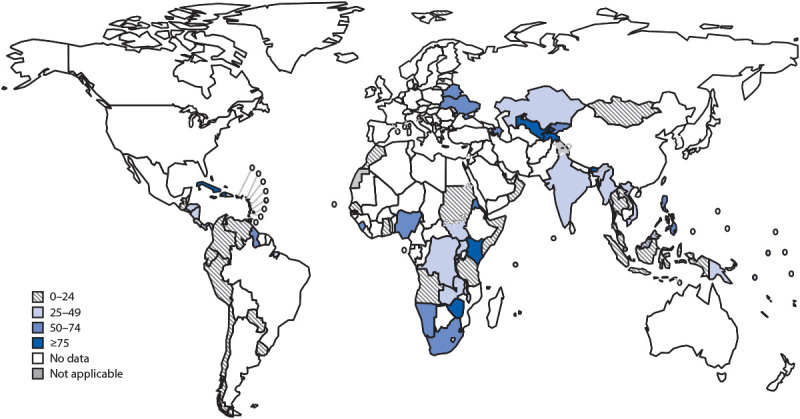
Percentage of persons living with HIV infection and on antiretroviral treatment who received tuberculosis preventive treatment — worldwide, 2019

**Source:** Adapted from World Health Organization. Global tuberculosis report 2020. Geneva, Switzerland: World Health Organization; 2020. https://www.who.int/teams/global-tuberculosis-programme/tb-reports

Among 17 countries that have completed national TB patient cost surveys since 2015, an average of 49% of TB-affected households faced catastrophic costs (country-level estimates = 19%–83%). This figure increased to 80% in households affected by drug-resistant TB (country-level estimates = 67%–100%) (*1*).

## Discussion

Globally, although TB incidence and mortality have been steadily decreasing, these declines are likely not occurring quickly enough for WHO End TB Strategy targets to be reached, with only a 9% decrease in incidence (2020 target = 20% decrease) and a 14% decrease in the number of deaths (2020 target = 35% decrease) from 2015 to 2019. The WHO European region is on track to reach both the incidence and mortality targets, and the African region has made progress toward meeting the targets. However, with one half of TB-affected households facing catastrophic costs, the world is far from reaching the WHO target of zero TB-affected households facing catastrophic costs.

Although challenges remain, assessment of the UNHLM-TB targets after the second year of the 2018–2022 timeline is more encouraging. During 2018 and 2019, a total of 14.1 million persons (35% of the UNHLM target) received TB treatment globally. For the target of 40 million patients treated to be achieved, an additional 26 million persons need to be treated during 2020–2022, which would represent substantial progress toward closing the gap between the number of persons who become ill with TB and the number who receive a diagnosis and are treated each year.

Although substantial progress has been made in TPT implementation, only 6.3 million persons, less than one fourth (23%) of the UNHLM-TB target, received TPT in 2018 and 2019. For the target of 30 million persons receiving TPT during 2018–2022 to be achieved, approximately 24 million additional persons must be reached with TPT during 2020–2022. Most persons who have received TPT to date are living with HIV infection, and the world is on track to reach the UNHLM target for this group. Despite strong growth in TPT provision to these persons, providing TPT to household contacts of TB patients, especially persons aged ≥5 years, continues to face substantial challenges.

Acceleration of TB service provision in 2020 was not possible in most countries because of the COVID-19 pandemic. Stay-at-home orders, movement restrictions, and the prioritization of COVID-19 mitigation activities have affected TB services through restricted service provision, diverted human resources, and disrupted supply chains (*6*). This has likely led to reductions in timely diagnosis and treatment of new tuberculosis cases (*7*). India, Indonesia, the Philippines, and South Africa reported monthly decreases in TB case notifications to approximately 50% of the January 2020 total during the first 6 months of 2020, with reductions of smaller magnitudes (25%–30%) reported by other high-incidence countries (*1*). The COVID-19 pandemic is continuing in 2021 and will have a long-term impact on national TB programs as well as global TB incidence and prevalence (*7*).

The findings in this report are subject to at least three limitations. First, underlying data quality, particularly for surveillance, might affect the accuracy of country estimates. Second, the differing methodologies used to generate country-level estimates might affect the comparability of estimates between regions and countries. Finally, a limited number of countries completed a national survey of costs faced by TB patients and their households, which might affect the generalizability of this indicator.

Programmatic efforts will need to be substantially enhanced for UNHLM targets for TB curative and preventive treatment to be reached by 2022, and more broadly, for future WHO End TB strategy targets to be met. For global TB targets to be achieved, innovations and adaptations in TB diagnosis, care, and treatment are needed to accelerate global TB progress and to meet the additional challenges presented by the COVID-19 pandemic (*8*), which threatens not only to slow future progress but also to reverse the gains made in recent years. However, the pandemic also provides new and unique opportunities to implement and evaluate innovations such as dual TB and COVID-19 screening of patients with respiratory symptoms, as well as multi-month dispensing of TPT and TB treatment combined with the use of digital health technologies to monitor patients in the context of fewer face-to-face encounters. Services for TB are an essential component of resilient health systems and can be strengthened by promoting synergies in the responses to both TB and COVID-19.

**Summary**

**What is already known about this topic?**

The 2018 United Nations High Level Meeting on Tuberculosis (TB) and the World Health Organization’s End TB Strategy set ambitious goals for reducing TB incidence, deaths, and patient costs and increasing the provision of TB curative and preventive treatment.

**What is added by this report?**

With an estimated 10 million incident TB cases and 1.4 million TB deaths in 2019, the world is not on track to achieve global targets. Further, the COVID-19 pandemic has hampered TB-related service delivery in many countries.

**What are the implications for public health practice?**

Innovations and adaptations in TB diagnosis, care, and treatment are needed to accelerate global TB progress and overcome the COVID-19 pandemic–associated challenges to TB diagnosis and treatment.
